# Supplementary material for: The use of virtual reality in screening for preclinical Alzheimer’s disease: A scoping review protocol
Source: PLoS One. 2023 Feb 27;18(2):e0282436. doi: 10.1371/journal.pone.0282436 (PMC9970083; doi:10.1371/journal.pone.0282436)
Supplement: Attachment — Submitted filename: Response to Reviewers.docx [file pone.0282436.s002.docx]

Dear Editor,

We would like to thank the reviewers for taking the time to provide such insightful feedback on the scoping review protocol that will undoubtedly improve the quality of the final review. We have provided our responses below which we hope to address the feedback provided sufficiently.

Please kindly let us know if you have any questions or more feedback about the response.

Best regards,

Yuan Tian, Maneesh V. Kuruvilla & Mira Park

| **Reviewer 1** | | |
| --- | --- | --- |
| No. | Reviewer’s comments | Authors’ response |
| 1. | I would like to see a more rigorous definition of VR in the introduction. For example, clarification on what you mean by 'screen-based' VR - there are articles that use 'VR' to describe participants looking at a monitor where physical movement (e.g. head rotation) does not correspond to digital movement updating. If VR is looking at a screen without physical-digital correspondence, then almost all cognitive papers examining preclinical AD can be included. This then becomes a scoping review protocol of preclinical AD, not VR. | Regarding the reviewer’s first comment related to the need for a more rigorous definition of VR, we now explicitly clarify the three types of VR, as outlined by Mazuryk and Gervautz (1996), that are distinguished based on the level of immersion offered to the user **(See *Manuscript* Lines 99-118)**. In clarifying the definition of VR, we describe how each of the three types of VR offer varying degrees of physical-digital correspondence. Regarding the reviewer’s second comment, we would like to begin by stating that, based on our chosen definition of VR, we would plan to include papers that use screens without physical-digital correspondence. As such, we agree with the reviewer that we will likely be able to include almost all cognitive papers examining preclinical AD. It is our opinion, though, that this does not restrict our protocol being either about preclinical AD or VR but rather about how technology, and specifically VR, is being used to develop cognitive biomarkers of preclinical AD. This is of immense relevance to researchers in both ICT and psychology/neuroscience. For example, if non-immersive VR - with no physical-digital correspondence - is sufficient to categorise individuals at-risk of AD, then this has huge benefits for the scalability and accessibility of a potential cognitive screening tool. Alternately, it may be the case that fully immersive VR - with supported physical-digital correspondence - is the need of the hour to accurately categorise individuals at-risk of AD given that a number of screening tools being developed involve aspects of orientation and navigation that are not realistically experienced on non-immersive VR. |
| 2. | In a similar vein, I would like to see a more rigorous definition of preclinical AD. Do these individuals have symptoms - should we consider them as having evidence of pathology or just modifiable/non-modifiable risk factors? This could further extend to detail about what biomarkers are considered sufficient evidence of preclinical AD. For example, Ab and tau positive PET/CSF evidence is stronger and more direct evidence of AD pathology than the presence of 1 or more ApoE4 alleles. | We agree that the protocol would benefit from a more rigorous definition of preclinical AD. We have, therefore, added a paragraph **(See *Manuscript* Lines 120-136)** applying a broad definition to preclinical AD that includes measures of positive pathophysiological markers (amyloid/tau) as well as high-risk factors (genetics/modifiable lifestyle factors etc.). |
| **Reviewer 2** | | |
| No. | Reviewer’s comments | Authors’ response |
| 1. | Introduction  “Spatial navigation is supported by brain areas that show some of the earliest signs of AD pathology”. I would add a sentence to spoke out these brain areas, at least mentioning the medial temporal lobe for example. | Noted and amended. “Spatial navigation is supported by structures in the medial temporal lobe, most notably the hippocampus and entorhinal cortex (9-11). The entorhinal cortex, in particular, shows some of the earliest signs of AD pathology (12), making tests of navigation an attractive option to assess preclinical AD over most established diagnostic tests of episodic memory (13).” **(See *Manuscript* Lines 45 - 49)** |
| 2. | “Tests of navigation often require larger-scale environments than can be set up and assessed in a small testing space [13] [14].”  I would change this sentence slightly to point out that there is virtual reality that simulate the environments as the small testing space here seems to refer to a simple tablet or a monitor. Something like “Tests of navigation often larger-scale environments, which can be fully simulated in VR, allowing for minimal testing space” | Noted and amended. “Tests of navigation often require larger-scale environments, which can be fully simulated in VR, necessitating minimal testing space (14, 15).” **(See *Manuscript* Lines 49-51)** |
| 3. | “The environments need to appear realistic but also allow for a degree of control to test specific aspects of navigation supported by brain areas in question [15] [16].”  After this sentence I would also introduce immersive Virtual Reality (iVR) which allows for more ecological valid studies to be conducted. Indeed, this added level of immersion adds to the realism of the environment, providing a more accurate representation of the space being tested. | Noted and amended. “Immersive VR, in particular, provides an opportunity to add to the realism of the environment thereby providing a more accurate representation of the space being tested and allowing for more ecologically valid studies to be conducted.” **(See *Manuscript* Lines 53-55)** |
| 4. | Table 2 I would add “computer” in Concept 1 keyword as sometimes you can find (especially in older literature) something like “computer-based test”. | **Response:** Noted and amended. The search term “computer” has been added under Concept 1 in Table 2 **(See *Manuscript* Line 155)**. |
| 5. | Table 3 It seems search #2 is missing the keyword “preclinical”. | **Response:** Noted and amended. Search #2 in Table 3 now includes the term “preclinical” **(See *Manuscript* Line 160)**. |
| 6. | Table 5 I would add an item which represent a category for the type of VR implied (desktop/semi/immersive). I have doubts about the definition of item 5. If I understand correctly item 5 would be used to outline if the study has tested also other population beside the inclusion criteria (preclinical AD) such as people diagnosed with AD. However, given the focus on the preclinical population and its importance as an inclusion criteria, I think it would be more helpful to add a separate item specifically for the preclinical population. This item could then be used to categorize how "preclinical AD" was assessed, such as by genetic risk and type, family history, or any other scoring methods. | Noted and amended. Items 7 and 8 have been added to Table 5 to capture what VR is used and how preclinical AD has been assessed respectively **(See *Manuscript* Line 205)**. |
| 7. | I like the wording of the types of VR implying the three types (desktop, semi, immersive). I think “semi” could be extended and by consequence an additional keyword in Table 3 1st row to AR (Augmented Reality). AR can be considered as specific class of “semi-immersive” and is gaining traction for being use as an assessment tool as the most ecological between all setups. | While we agree with the reviewer that AR offers significant potential as an ecologically valid digital-physical world interface, it is our view that the inclusion of AR is beyond the scope of the current protocol in terms of the way VR has been defined. The chosen definition of VR, irrespective of the level of immersion offered, encapsulates a user experience involving a fully digital environment. Given that AR offers an overlay of the virtual environment on to the physical one, we believe that including AR in the search strategy is beyond the scope of the current protocol. |
| 8. | I don t think that there are many studies looking at preclinical AD so far, so I would be very cautions with keywords and exclusion criteria. For example, in Table 2 Concept 3 I would add also “evaluating”, “testing”, “analysing”. Connected to this Table 4 exclusion criteria might be too strict. I would simplify by removing point 2 and 4 and I would make sure to include synonyms to the word “screening” for point. My concern is excluding a study that think of VR as a very secondary part of the study and thus mention the technique only in the methods. | Noted and amended. Synonyms for “screening” have been added under Concept 3 in Table 2 **(See *Manuscript* Line 155).** Additionally, the exclusion criteria in Table 4 have been simplified **(See *Manuscript* Line 183)**. |

**For your information, we have also made additional changes to improve the protocol, building on the above-mentioned modifications.**

| No. | Other changes |
| --- | --- |
| 1. | To make it convenient for potential readers to check the online registration, we have put down the URL of the OSF registration (<https://osf.io/aqmyu>), instead of the project URL (<https://osf.io/djrky/>) **(See *Manuscript* Line 37)** |
| 2. | Since the search strategy has evolved based on additional keyword recommendations by Reviewer 2, the processes to identify eligible studies **(See *Manuscript* Lines 170 - 177)** and to chart the data **(See *Manuscript* Lines 186 - 191)** have been modified, considering the large volume of potentially relevant records. |
| 3. | Figure 1, the flow diagram, has been updated. **(Fig1.tif)** |
| 4. | The Preferred reporting items for systematic review and meta-analysis protocols (PRISMA-P) checklist has been updated, as per the modifications of the manuscript. Specifically, we have replaced the (PRISMA-P) checklist with the latest (PRISMA-ScR) checklist, which is relevant for scoping reviews. **(S1 Table 1)** |
| 5. | Senior authorship has been specified. **(See *Manuscript* Line 12)** |

Authors: Yuan Tian, Maneesh V. Kuruvilla and Mira Park

Date: January-10-2023
